# Significance of cutting plane in liquid metal embrittlement severity quantification

**DOI:** 10.1007/s42452-021-04608-2

**Published:** 2021-05-10

**Authors:** C. DiGiovanni, L. He, C. Hawkins, N. Y. Zhou, E. Biro

**Affiliations:** Department of Mechanical and Mechatronics Engineering, University of Waterloo, Waterloo, ON Canada

**Keywords:** Resistance spot welding (RSW), Liquid metal embrittlement (LME), Metallographic preparation, Quantitative metallography

## Abstract

The automotive industry is turning to advanced high strength steels (AHSS) to reduce vehicle weight and increase fuel efficiency. However, the zinc coating on AHSS can cause liquid metal embrittlement (LME) cracking during resistance spot welding. To understand the problem, the severity of the cracking must be measured. Typically, this is done from the weld cross-section. Currently, there is no standard procedure to determine which plane through the weld must be examined to gauge cracking severity, leading to a variety of practices for choosing a cutting plane. This work compares the magnitude and variability of LME severity measured from the plane of exhibiting the most severe surface cracking to arbitrarily chosen planes. The plane exhibiting the most severe cracks had more and longer cracks on the cross-section than the arbitrarily chosen plane, resulting in a higher crack severity measurement. This higher absolute measurement increased the relative accuracy of the examination, allowing for fewer welds to be examined to precisely determine the effect of LME mitigation methods on cracking severity, how welding parameters affect LME cracking severity and the predicted LME affected strength of a particular weld.

## Introduction

Advanced high strength steel (AHSS) is being adopted to increase material strength for automotive parts. However, when some of these steels are resistance spot welded (RSW) during automotive assembly, the protective zinc coating on the steel surface melts and enters the grain boundaries of the steel substrate, leading to intergranular cracking [1–3]. This is known as liquid metal embrittlement (LME), and may decrease spot weld strength [4–6].

Due to concerns about the impact of LME cracks on RSW strength, automotive standards are strict on how large surface cracks may be before welds are no longer fit-for-service [7]. It should also be noted that no cracks in the weld shoulder are permissible, which is where LME cracks are often seen are seen [3]. These standards are in-line with research that shows that the depth of shoulder cracks can affect the degree of strength loss exhibited by welds with LME cracks when compared to uncracked welds [8, 9]. Furthermore, these studies showed LME shoulder cracks less than 300–325 μm (for these specific joints) did not affect joint strength.

Precisely correlating crack depth to weld strength degradation for a particular weld is unrealistic because there is large weld-to-weld variation in LME crack severity and location [10], and in a manufacturing setting, there are insufficient resources to measure crack depths from all welds. However, considering that a particular combination of welding parameters will result in a repeatable depth/quantity distribution of LME cracks in multiple welds [6, 11], cracking severity may be classified based on the number and depth of the entire population of cracks found in a particular weld. This approach was used by Wintjes et al*.* [6] to correlate an LME cracking severity index to welds strength degradation.

With the understanding of how LME crack dimensions and quantity can affect weld strength, as well as how LME cracks may be characterized in a repeatable way, weld crack measurements may be used to optimize the welding process by minimize cracking, and gain further understanding of how crack orientation and loading configuration affect joint strength [9]. However, to do this, a method to measure cracking severity must be determined. The only way to measure the entire population of cracks within a weld is by using volumetric techniques such as x-ray computer tomography (CT) scanning [12, 13], or using serial metallography where the specimens are repeatedly ground a finite amount and successive planes are inspected for cracks [14]. However, these techniques are very time intensive and are not well suited to characterizing LME due number of replicates needed to accurately measure crack distribution because of high weld-to-weld variability. Excluding volumetric cracking measurement means that planar measurement is the only viable option to characterize cracking distribution. Many studies have assessed cracking susceptibility from plan view (as seen from the top of the weld) using methods such as optical microscopy [1, 15, 16], liquid dye penetrant [2, 17], radiography [18], fluorescent magnetic particle detection [19] and pulsed eddy current thermography [20]. However, using these techniques only views the weld surface. No information about crack depth may be learned, which gives no insight into whether the observed LME cracks will affect weld strength. Therefore, to balance testing efficiency and insight into post-welding properties, LME cracks must be measured from the weld cross-section.

For the reasons stated above, many studies have measured LME cracking severity from the weld cross-section. However, in the literature there seems to be two major philosophies on choosing the cross-section plane; these are the plane intersecting the most severe cracks [1, 6, 19, 21–23], and a plane with a fixed orientation to the welding coupon [16, 24, 25]. In the above cases the cross-section plane intersected the center of the weld (lying along the weld diameter). Although, it should be noted that in many cases, the method used to determine the cross-section plane was not described [2, 26, 27], and in one case, the cross-section plane did not necessarily bisect the weld [28]. Considering the importance that LME crack dimensions has on weld properties, it is important to understand how measurements taken from a particular cross-section plane are representative of the crack distribution for a set of welds made with a single set of welding parameters. Furthermore, considering the large amount of research in this area, it must be known if results from various studies may be compared. This study examines the distribution of LME cracks in a set of welds made with standard test of welding conditions as measured from both cross-sections planes intersecting the most severe surface crack and those that are chosen randomly. The cracking severity from both measurements are compared in terms of crack depth and frequency, and recommendations are put forward as how weld cross-sections should be prepared for analysis to ensure the most representative results while minimizing testing effort.

## Materials and methods

A transformation induced plasticity steel with a tensile strength of 1100 MPa (TRIP1100) and thickness of 1.6 mm was used in this study. This material is susceptible to LME cracking and is of interest to industry. The chemical composition of the steel may be found in Table 1. Twelve samples were welded using a DC pedestal spot welder with an air over oil force actuation system. All welds were made at the expulsion current, with the remaining welding parameters were selected from AWS D8.9 [29] and are listed in Table 2.

**Table 1:** Chemical composition (wt%) of examined steel

C	Mn	Si	Al	Cr	Mo	Fe
0.20	2.2	1.6	–	–	–	Bal.

**Table 2:** Welding parameters in accordance with AWS D8.9 [29]

No. of pulses	Current (kA)	Weld time (ms)	Cool time (ms)	Force (kN)	Hold time (ms)
2	9.5	200	33	5.5	167

**Fig. 1:**
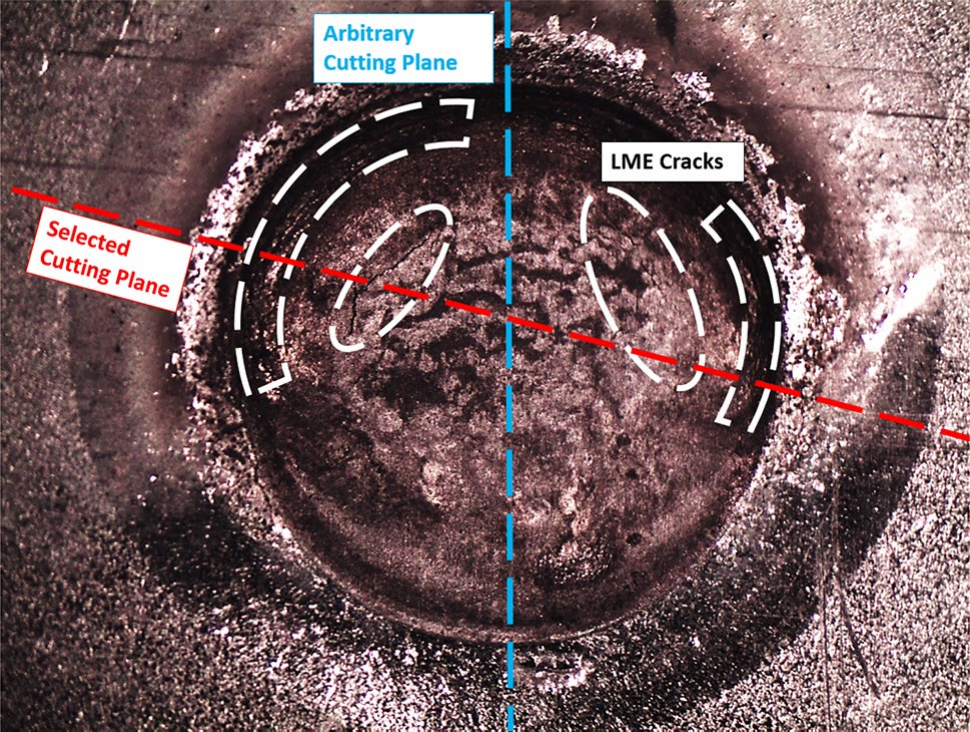
Cutting planes using the selected cutting plane and arbitrary cutting plane methods

After welding, six samples were randomly chosen and cross-sectioned along arbitrary cutting planes through the center of the weld nugget. This method of plane selection is called the arbitrary cutting plane method. The remaining six samples were first inspected for surface cracks using a stereomicroscope, then the cutting planes were chosen so that they would intersect through both the center of the weld nugget and the greatest number of surface cracks, as shown in Fig. 1. This is referred to as the selected cutting plane method.

**Fig. 2:**
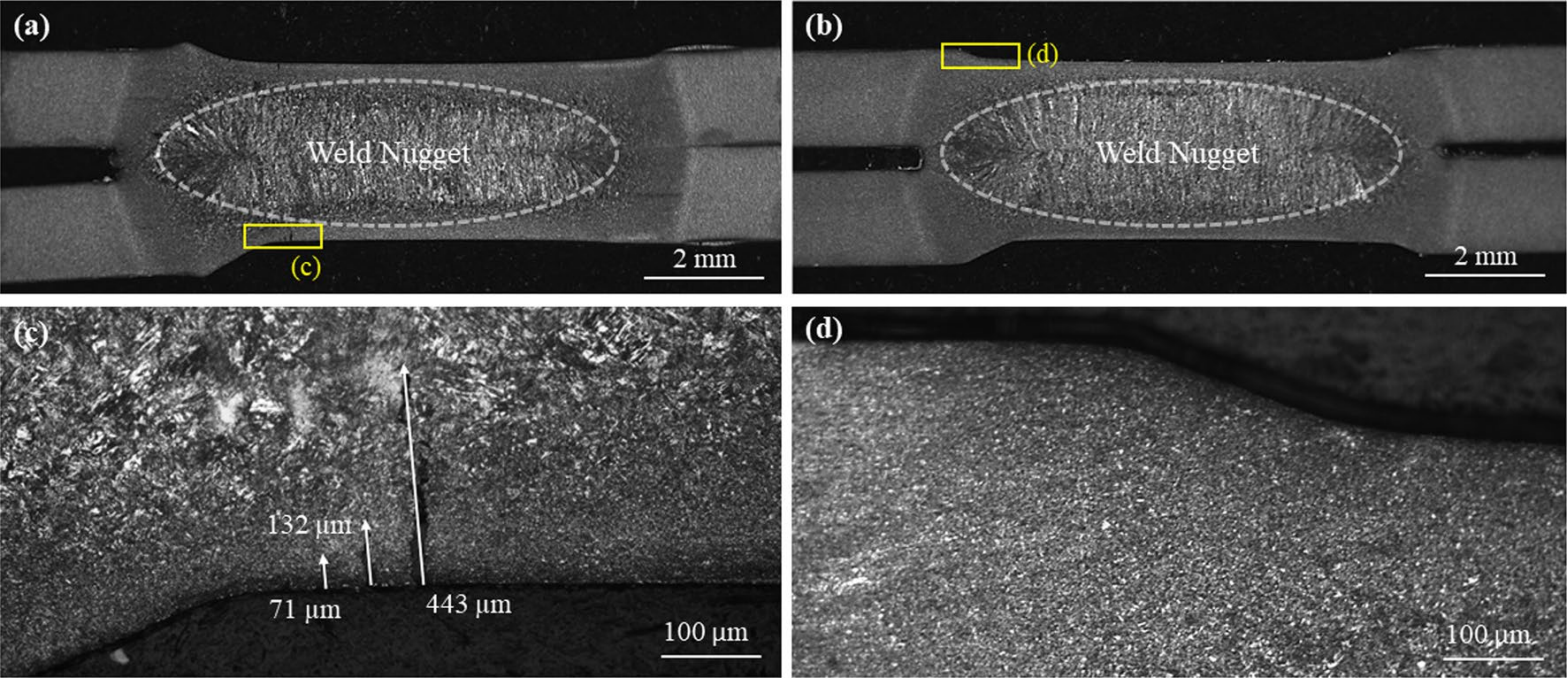
Examples of cross-sections of cracked welds using the **a** selected and **b** arbitrary cutting plane methods, with increased magnification images of the weld shoulder from cross-sections **c** using the selected and **d** arbitrary cutting planes. Examples of how crack depth was measured may be seen on inset (**c**)

After cross-sectioning, all samples were hot mounted, ground, polished to a 1 μm diamond finish using standard metallographic techniques. After polishing the cross-sections were rinsed with methanol and etched with 5% nital for 2 s to reveal the weld nugget. An optical microscope at 200 × optical magnification was used to observe all revealed LME cracks on the cross-section and the crack depths were measured using ImageJ® software. Crack depth was measured as the straight line distance from the crack opening to the crack root as per previous work [6] and shown in Fig. 2. After the cracks in each weld cross-section were measured, cracking severity was calculated using the cracking index (CI), a measure that combines the depth and number of cracks observed from a particular weld into one value that has been shown to relate to strength loss in spot welds pulled in tensile shear [6]. The cracking index is defined in Eq. (1)

1 $$CI{ } = { }\frac{nL}{\tau }$$

where *n* is the mean number of measured cracks per sample, *L* is the lognormal median (geometric mean) crack depth, and τ is the sheet thickness. A higher crack index indicates more severe LME cracking. Standard statistical methods were used to test the data. The specific tests are noted in the Results and Discussion section. The standard deviation associated with the lognormal median crack length was calculated using the bootstrap method with 250 datasets of the same number of values as the original measurements; 61 and 24 for the cases of the selected and arbitrary cutting planes, respectively. All error bars represent a 95% confidence interval and all confidence tests were calculated using a 5% significance level.

## Results and discussion

### Distribution of cracks and LME severity measurements

**Fig. 3:**
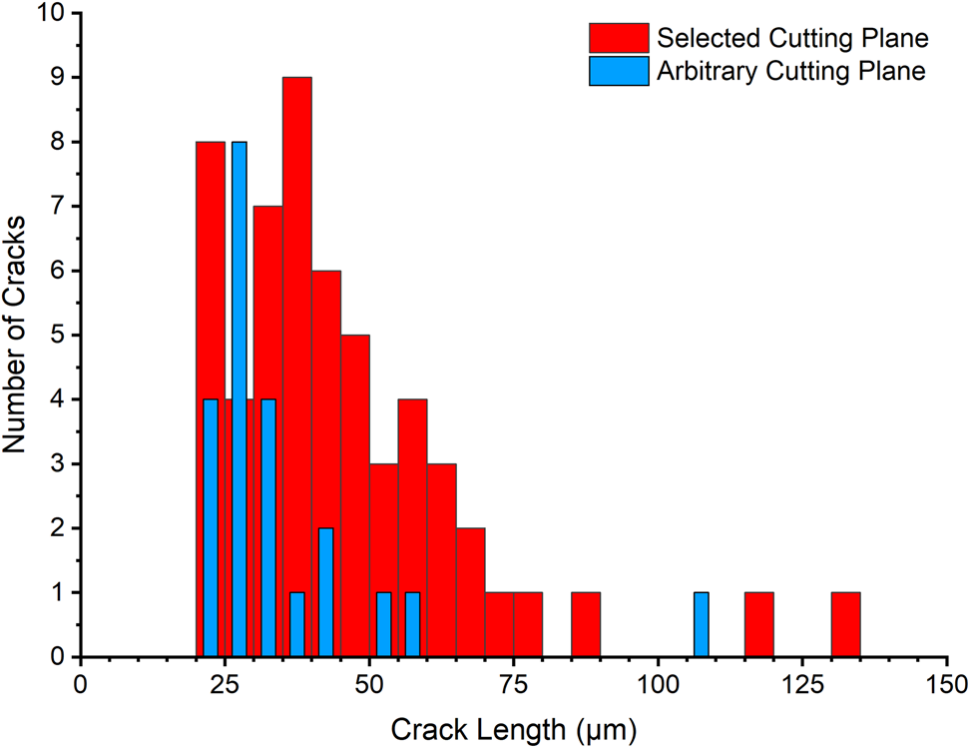
Histogram of crack distribution measured from cross-sections chosen using both the selected and arbitrary cross-section planes

The distribution of cracks measured from the cross-section of both welds were compared and it was immediately seen that the selected cutting plane revealed more cracks, and the observed cracks were deeper than seen on the arbitrary cutting plane (see Fig. 3), which agrees with the qualitative observations of the cross-sections in Fig. 2. The crack distribution from the selected cutting plane also showed a higher number of small cracks < 25 μm as well as much larger cracks. Cross-sections from the selected cutting planes contained 5 cracks that were deeper than 150 μm (truncated from Fig. 3), whereas only 1 crack deeper than 150 μm was seen from observations made on the arbitrary cutting plane.

**Fig. 4:**
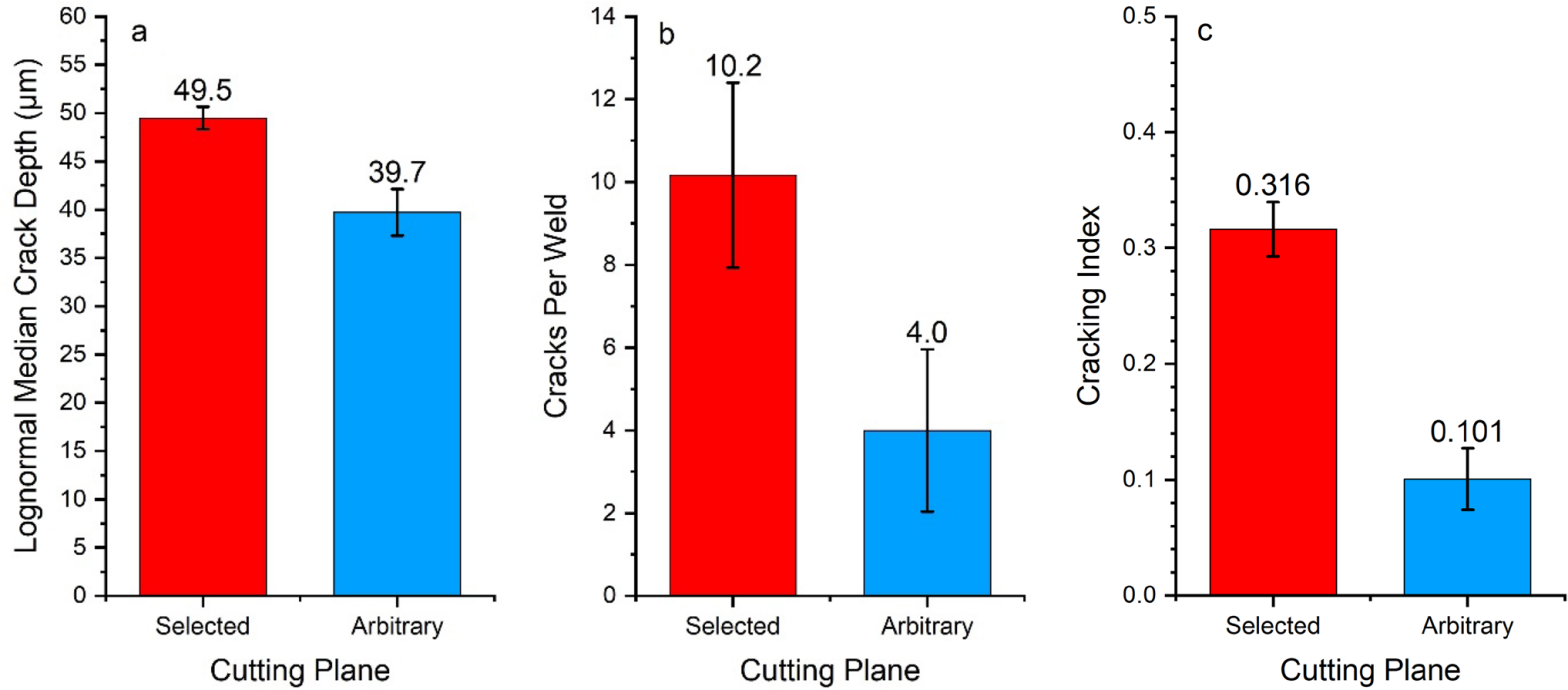
Comparison of the **a** lognormal median of measured cracks depths, **b** cracks per welds and **c** cracking index measured from welds cross-sectioned from both selected and arbitrary cross-sectioning planes

To determine the cracking severity, the lognormal median crack depth and cracks per welds were calculated from both sets of cross-sections. Similar to the crack distribution (Fig. 3), the cracks measured from the selected cutting plane were both deeper on average and more numerous than cracks observed from the arbitrary cutting plane (see Fig. 4). The cracks observed from the selected cutting planes had an average crack depth of 49.5 µm and the welds contained an average of 10.2 cracks per weld (61 cracks total), whereas the arbitrary cutting planes showed 4.0 cracks per weld (24 cracks total), which had an average depth of 39.7 µm. When compared using a T-test, it was seen that both differences in measured crack depth and crack frequency measured using the two methods were significantly different. It should also be noted that the distribution of cracking data (standard deviation) of the crack depth measurements calculated from the selected cutting plane was significantly narrower than that calculated from the cracks measured from the arbitrary cutting planes (see Table 3), as determined by the F-Test (significance value of 0.002). This had the effect of increasing the confidence interval size for the average depth of cracks measured from the arbitrary cutting plane, decreasing the certainty of average (wider error bars).

**Table 3:** Uncertainty associated with LME cracking metrics from welds prepared using the selected and arbitrary cutting plane methods

Cross-sectioning methodology	Degrees of freedom	Log normal median crack depth (μm)		Cracks per weld		Cracking index	
		Standard deviation	95% confidence interval	Standard deviation	95% confidence interval	Standard deviation	95% confidence interval
Selected cutting plane	60	4.0	1.0	2.8	2.2	0.091	0.024
Arbitrary cutting plane	23	6.8	2.7	2.4	2.0	0.063	0.027

The cracking index for the welds were calculated from the crack depth and frequency data from each of the cross-sections using Eq. (1). As would be expected, the selected cutting plane had a much higher cracking index (0.32) than the arbitrary cutting plane (0.10), as may be seen in Fig. 4c. This was due to the arbitrary cutting plane having fewer and shallower cracks than found on the selected cutting plane. It should be remembered though that all welds were made using the same welding conditions, so the LME crack distribution throughout each weld, both observed and unobserved, should be equivalent. However, the difference in the measured LME severity index between the two observed planes shows that there is a much higher density of cracks below the large visible surface cracks than may be found on arbitrary cross-section planes. This is logical as LME cracking initiates from the material surface when Zn from the coating degrades the surface grain boundaries [30]. Therefore, cracks must be open to the surface. It stands to reason that neither cross-sectioning technique provides a true picture of the cracking distribution within the weld, but it is also logical that the selected cutting plane represents a worst-case scenario of the local crack distribution.

Although the CI combines crack depth and crack frequency in single measurement [Eq. (1)], it is interesting to note that even with higher uncertainty (confidence interval) associated with the crack depths measured from the arbitrary cutting plane than the selected cutting plane, there was not a significant difference between the uncertainty of the CI results associated with the two cutting planes. This is most likely due the large uncertainty associated with the crack frequency measurements (Fig. 4b) and the fact that when multiplying numbers with associated uncertainties, part of the calculation to determine the resultant uncertainty is multiplying the variance by the multiplied quantity (lognormal median depth and cracks per weld in this case). As the multiplied quantities for the arbitrary cutting plane were small compared to those of the selected cutting plane, the resultant uncertainty was also small. However, even though the uncertainties associated with the two planes are similar, it should be noted that the due to the lower CI value resulting from the arbitrary cutting plane, the uncertainty is a much larger fraction of the total cracking severity measurement. In the case of the data from the arbitrary plane, the confidence interval represents 27% of the CI measurement, whereas it is only 8% in the case of the CI measurements from selected plane. This large relative uncertainty associated with the arbitrary cross-section crack data will have large implications on the ability to make decisions from this data.

### Influence of cutting plane on improving measurement precision

**Fig. 5:**
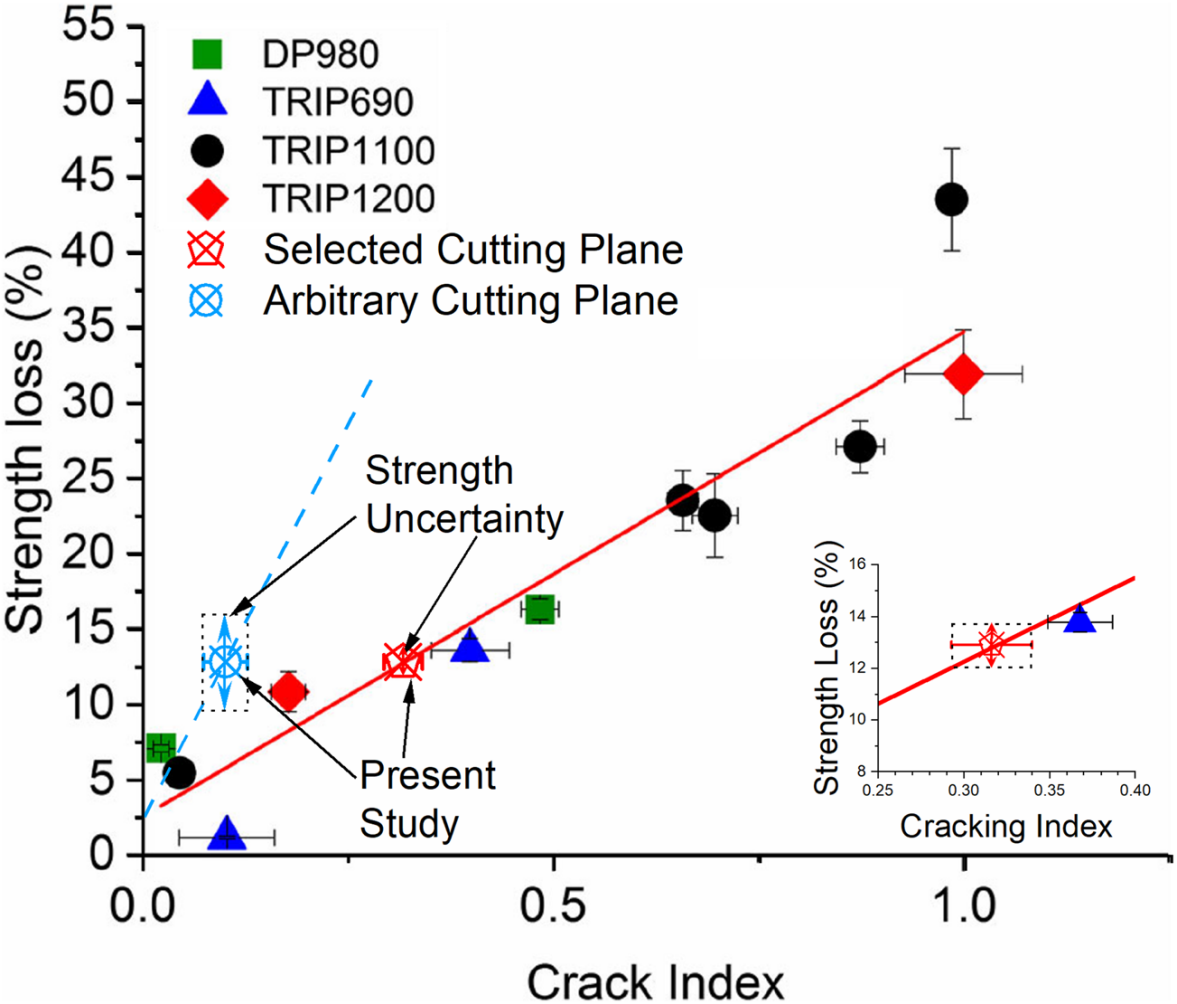
Correlation between cracking index and percentage tensile shear strength loss for various steels with data included from the present study, with an inset showing a higher resolution view of points from cracking index values from 0.25 to 0.40. Adapted from [6]

As LME cracking has been shown to affect tensile shear strength [5, 8, 9] and the drop in strength may be predicted from LME cracking data [6], it is important to have precise knowledge of the LME cracking conditions within a weld. To understand the how the CI data calculated from the measurements taken from the selected and arbitrary cutting planes impacts interpretation of post-weld properties, the data presented in Fig. 4c was compared to the correlation Wintjes et al. [6] made between weld strength loss and CI (Fig. 5). The data from the present study was plotted on Fig. 5 under the assumption that the selected plane data would have a strength loss predicted by Wintjes et al. [6], as both studies used similar welding methodologies and the cross-sectioning technique used by Wintjes et al. was similar to the selected cross-section method, and the arbitrary cutting plane data should exhibit the same strength loss exhibited by the selected cutting plane data, as all welds were made using the same welding parameters. The strength uncertainty was calculated as the predicted strength values associated with the cracking index values at the extreme ends of the 95% confidence interval of both data points, as shown by the dotted box around the points associated with the present study. By plotting these two points, it may be seen that both methodologies should follow separate strength-loss/crack index correlations, assuming the linear trend seen for the selected cutting plane is also true for arbitrary cutting planes. However, due to the higher relative uncertainty of the CI measured from the arbitrary planes there is also less certainty about the strength loss exhibited by the joint. The predictions of percentage of post-weld strength loss when compared to an uncracked weld made from the CI value calculated using the selected cutting plane data is accurate to ± 0.8%, whereas the CI value calculated from the arbitrary cutting planes can only predict strength loss within ± 2.8%. The total range of the uncertainty in the case of the arbitrary selected cutting planes represents almost 50% of the predicted strength loss value. The strength loss uncertainty is shown by the vertical arrows in the Fig. 5; an inset is added to clarify the uncertainty associated with measurements from the selected cutting planes. As indicated earlier, the selected plane method of LME crack analysis may provide a worst-possible case of local cracking distribution, however, this example shows how exaggerating the measured cracking severity helps to improve measurement resolution. In this case, the exaggerated cracking metric improved mechanical property predictions, but it may also be used to improve resolution when understanding how welding parameter changes affects LME crack severity.

**Fig. 6:**
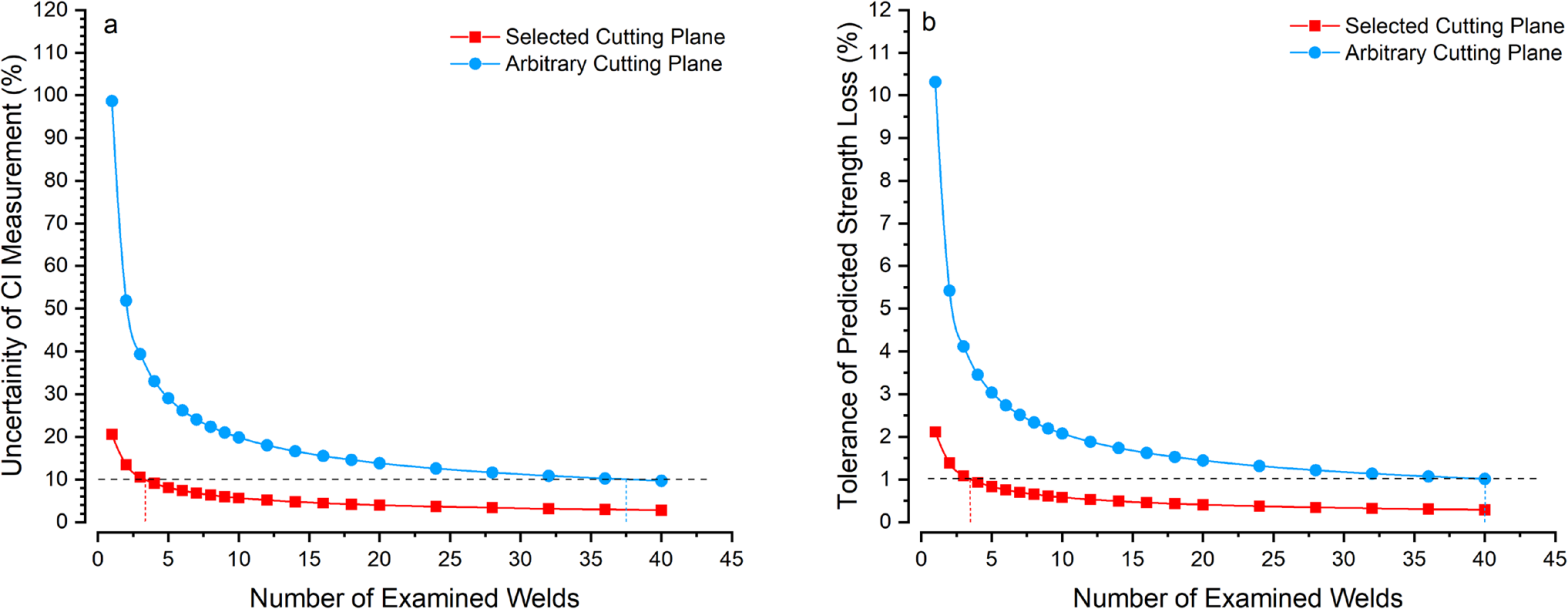
Predicted uncertainty associated with **a** cracking index calculation and **b** tensile shear strength loss associated with LME cracking from the number of welds examined to determine weld cracking severity

The use the selected cutting plane to measure the most severe local cracking in the weld will also decrease the necessary number of welds to be examined to ensure accurate results. The level of certainty of the predicted cracking severity (CI) depends on two things: the standard deviation of the measurements, and the number of cracks measured. Although the standard deviation of the CI calculation using the selected cutting plane method is higher than that for the CI using the arbitrary plane method, 0.091 and 0.063, respectively (see Table 3), the selective cutting plane method revealed about 2.5 times the number of cracks compared to the arbitrary cutting plane method. This means that the averages used to calculate the CI are based on a greater number of measured cracks, so the associated uncertainty decreases. From cracks in the present study it may be seen that using the selected cutting plane method drastically reduces the number of cross-sections needed to precisely predict cracking severity. Using the welds in the present study as an example, when the selected cutting plane method is used to reveal LME cracks, only 4 welds are needed to calculate CI with a confidence interval of ± 10%, whereas using the arbitrary cutting plane method 38 welds are needed (see Fig. 6a). If strength degradation is being calculated, then prediction becomes even more accurate as the slope of the strength-loss CI curve in Fig. 5 is shallow for LME cracks measured using the selected cross-section plane. In this case, analysing 4 welds cross-sectioned using the selected cutting plane will enable of a strength loss prediction of ± 1%, whereas 40 welds are needed to calculate strength loss to the same precision using the arbitrary cutting method (see Fig. 6b).

## Conclusions

With the large amount of work being done to understand and reduce LME in the automotive sector, there have been many methodologies used to characterize cracking severity. Due to the need to understand crack depth and carry-out cracking analysis in a time-efficient manner, the majority of LME crack characterization is carried out from weld cross-sections. However, this requires researchers to choose where to cross-section the weld. The current study compared the common methods used to choose the cross-sectioning plane for crack analysis: the selected method, chosen to intersect the largest crack, and the arbitrary method, chosen at random without regard to the location of surface cracks. It was found that analyzing LME cracks using the selected cutting plane was far superior to the arbitrary cutting plane. The selected cutting plane revealed more and deeper cracks than were found on the arbitrary cutting plane. This resulted in a higher cracking severity measurement with a lower relative uncertainty. The lower relative uncertainty enabled higher measurement precision, needing fewer samples to examine to achieve acceptable results. Furthermore, this study showed that crack distribution in the weld is not uniform. Although some work may seek to understand the global cracking distribution in the weld, it is thought that the worst-case-scenario measured using the selected cutting plane not only results in more conservative results when predicting the effect of LME cracking on post-weld performance, but it also allows a higher measurement resolution, requiring fewer samples to be analyzed to understand how crack reduction methodologies affect LME cracking severity. Adoption of the selected cutting plane to analyze LME cracking will improve the resolution of LME crack measurements. This will better show how improved materials and weld procedures that are being tested with the objective of reducing LME are effective, which will reduce the time needed to address this issue. This will, in-turn, speed the adoption of high strength AHSS into automotive designs, leading to the associated decreases in vehicle emissions and increases in passenger safety.
